# The complete plastome sequence of *Lilium Bulbiferum* (Liliaceae)

**DOI:** 10.1080/23802359.2017.1419090

**Published:** 2017-12-20

**Authors:** Hyoung Tae Kim, Ki-Byung Lim

**Affiliations:** a Department of Horticulture, Kyungpook National University, Daegu, Republic of Korea;; b Institute of Agricultural Science and Technology, Kyungpook National University, Daegu, Republic of Korea

**Keywords:** *Lilium bulbiferum*, plastome, phylogeny

## Abstract

The flower shape of *Lilium bulbiferum* L. is different from that of the other European lilies. The phylogenetic position of the plant has been changed frequently. In the present study, we have sequenced the complete plastome of *L. bulbiferum*. The plastome sequence was 152,686 bp long, with a large single copy (LSC) region of 82,083 bp, a small single copy (SSC) region of 17,619 bp, and two inverted repeat (IR) regions of 26,492 bp each. It contained 133 genes, including 83 coding genes, 8 ribosomal RNAs, 38 transfer RNAs, and 4 pseudogenes. The GC contents of LSC, SSC, and IT were 34.8%, 30.7%, and 42.5%, respectively, corresponding to 37.0% of the total GC content. The phylogenetic position of *L. bulbiferum* was closely related to the north-eastern lilies.

*Lilium bulbiferum* L. is native to Europe, and is a rare plant in the wild (McRae et al. 1998; İkinci et al. 2006). This species is thus named because of the numerous bulbils in its leaf axils (McRae et al. 1998). Because of its erect bowl-shaped flower, Baker (1871) and Wilson (1925) distinguished *L. bulbiferum* from the other European lilies. However, Comber (1949) reclassified the genus *Lilium*, and placed *L. bulbiferum* under the section *Liriotypus*, which included all European lilies, except *L. martagon*. Although many phylogenetic studies have been conducted on the genus *Lilium*, the phylogenetic position of *L. bulbiferum* remains unclear owing to its low phylogenetic resolution (Nishikawa et al. 2001; İkinci et al. 2006).

The complete plastome of *L. bulbiferum* was sequenced in the present study. Genomic DNA was extracted from the fresh leaves of *L. bulbiferum* by CTAB method (Doyle 1987), and it is stored in Kyungpook National University. Total DNA was sequenced using a HiSeq 2500 instrument (Illumina, San Diego, CA). The assembly strategy for mapping the plastome sequence followed the method of Kim et al. (2015). The average depth of the plastome sequence was 458.2, with maximal and minimal depths of 759 and 152, respectively.

Gene annotation of plastome was performed using Geneious (Kearse et al. 2012) by comparing with the plastome sequences of other *Lilium* species. For phylogenetic analysis, plastome sequences of 20 *Lilium* and 3 *Fritillaria* species were downloaded from the NCBI data base, and a total of 77 genes were extracted from their plastome sequences. Each gene was aligned by MAFFT (Katoh et al. 2002), and the alignments were concatenated. The phylogenetic tree was constructed using RAxML (Stamatakis 2014) with GTR + G + I model in the CIPRES Science Gateway (Miller et al. 2010).

The plastome sequence of *L. bulbiferum* (GenBank accession number MG574829) was 152,686 bp long, with a large single copy (LSC) region of 82,083 bp, a small single copy (SSC) region of 17,619 bp, and two inverted repeat (IR) regions of 26,492 bp each. It contained 133 genes, including 83 coding genes, 8 ribosomal RNAs, 38 transfer RNAs, and 4 pseudogenes. Interestingly, the *psaJ* gene was extended by six bp, since its stop codon sequence TGA was substituted with TTA. The GC contents of LSC, SSC, and IR were 34.8%, 30.7%, and 42.5%, respectively, corresponding to 37.0% of the total GC content.

The phylogenetic analysis using 21 *Lilium* and 3 *Fritillaria* species showed that the genus *Lilium* can be distinguished into two main groups and *L. bulbiferum* was a sister clade of the north-eastern lilies (Figure 1). The plastome sequence of *L. bulbiferum* is the first to be reported among the European lilies. Further, we expect that this sequence will help clarify the origin of European lilies, as well as new circumscription of the genus *Lilium*.

**Figure 1.**
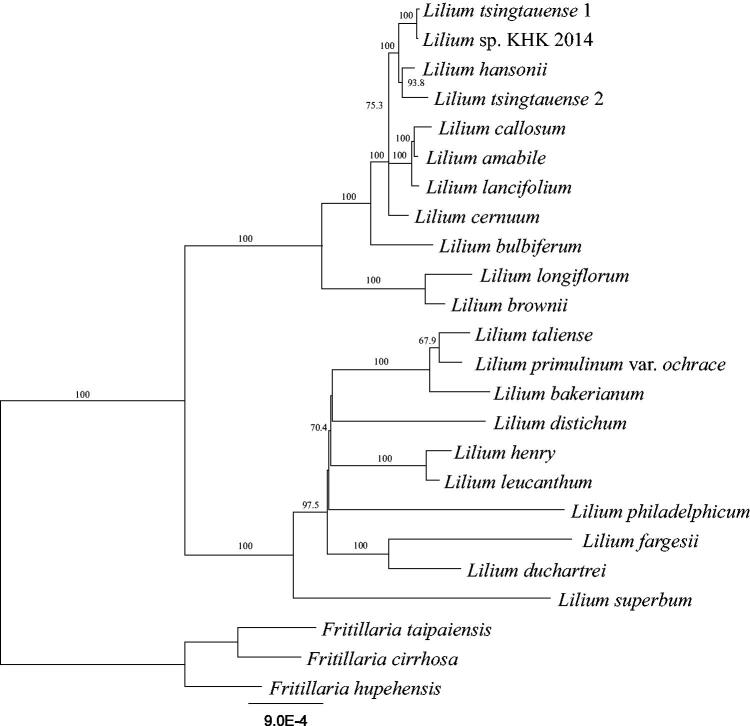
Maximum likelihood phylogenetic tree using 77 genes from 21 *Lilium* and 3 *Fritillaria* species. The *Fritillaria* species were used as outgroup. The numbers on the node refer to bootstrap values. Scale refers to substitutions per site.
